# Combined systemic immune-inflammatory index (SII) and prognostic nutritional index (PNI) predicts chemotherapy response and prognosis in locally advanced gastric cancer patients receiving neoadjuvant chemotherapy with PD-1 antibody sintilimab and XELOX: a prospective study

**DOI:** 10.1186/s12876-022-02199-9

**Published:** 2022-03-14

**Authors:** Ping’an Ding, Honghai Guo, Chenyu Sun, Peigang Yang, Na Hyun Kim, Yuan Tian, Yang Liu, Pengpeng Liu, Yong Li, Qun Zhao

**Affiliations:** 1 The Third Department of Surgery, The Fourth Hospital of Hebei Medical University, Shijiazhuang, 050011 Hebei China; 2 Internal Medicine, AMITA Health Saint Joseph Hospital Chicago, 2900 N. Lake Shore Drive, Chicago, IL 60657 USA

**Keywords:** Systemic immune-inflammatory index (SII), Prognostic nutritional index (PNI), Chemosensitivity, Immunotherapy, Locally advanced gastric cancer

## Abstract

**Background:**

Previous studies have confirmed that systemic immune-inflammatory index (SII) and prognostic nutritional index (PNI) can predict the prognosis and chemotherapy efficacy of various malignant tumors. However, to the best of our knowledge, no study investigated the SII combined with PNI score to predict the efficacy of anti-programmed death 1 (anti-PD-1) antibody sintilimab and XELOX regimen (capecitabine plus oxaliplatin) in the treatment of locally advanced gastric cancer. This study aims to evaluate the predictive value of pre-treatment SII-PNI score on the sensitivity of sintilimab immunotherapy combined with XELOX chemotherapy in patients with locally advanced gastric cancer.

**Methods:**

We registered a prospective clinical study involving 30 locally advanced gastric cancer patients from March 2020 to July 2021. The pre-treatment SII and PNI were calculated from peripheral blood samples, and the cut-off value was calculated by receiver operating characteristic. The SII-PNI score ranged from 0 to 2 and were categorized into the following: score of 2, high SII (≥ 568.5) and low PNI (≤ 52.7); score of 1, either high SII or low PNI; score of 0, no high SII nor low PNI.

**Results:**

All patients were evaluated by RECIST1.1 criteria after four cycles of sintilimab immunotherapy combined with XELOX chemotherapy, including 5 patients with TRG 3 and 25 patients with non-TRG 3. The SII-PNI score of non-TRG 3 patients was significantly lower than that of TRG 3 patients (*P* = 0.017). The medial progression free survival of patients with low SII-PNI score was significantly better than that of patients with high SII-PNI score (*P* < 0.001). Multivariate analysis showed that SII-PNI score was an independent prognostic factor for predicting progression-free survival (*P* = 0.003).

**Conclusion:**

The pre-treatment SII-PNI score is a significant indicator for predicting chemosensitivity of locally advanced patients after sintilimab immunotherapy combined with XELOX chemotherapy, which can help to identify high-risk groups and predict prognosis.

*Trial registration*: The registered name of the trial is “Prospective clinical study of sintilimab combined with chemotherapy for neoadjuvant therapy in locally advanced gastric cancer”. Its Current Controlled Trials number is ChiCTR2000030414. Its date of registration is 01/03/2020.

## Introduction

Gastric cancer is one of the most common malignant tumors of digestive tract, with high morbidity and mortality [1]. In China, the majority of gastric cancer patients were in advanced stage at the time of diagnosis [2]. The 5-year survival rate of patients with advanced gastric cancer is around 40%; those without surgery only have approximately 10% 5-year survival rate [2]. At present, the main treatment options for locally advanced gastric cancer include surgery, chemotherapy, radiotherapy, targeted therapy and immunotherapy [3]. Our previous study found that neoadjuvant chemotherapy of XELOX regimen for locally advanced gastric cancer can effectively improve the resection rate of R0 surgery and improve the prognosis [4]. In addition, anti-programmed cell death 1 (PD-1) antibodies, such as nivolumab, sintilimab, and camrelizumab, have been shown to improve overall survival (OS) and progression-free survival (PFS) in locally advanced gastric cancer patients [5–7]. Unfortunately, not all patients can achieve better chemotherapy efficiency and benefit from those treatments. Studies have shown that factors that may affect the sensitivity of chemotherapy include tumor differentiations or certain gene expressions [8, 9]. However, there is still a lack of reliable indicators to predict tumor response and prognosis of patients before chemotherapy, and to ultimately optimize treatment strategy.

Accumulating evidence has revealed that the occurrence and development of gastric cancer are closely related to the tumor inflammatory microenvironment [8, 10]. The Systemic Immunoinflammatory Index (SII) is a novel inflammatory index calculated based on peripheral blood neutrophils, platelets and lymphocytes, which can represent different inflammatory and immune pathways in vivo and has greater stability [11]. Numerous studies have shown that SII can predict the prognosis of malignant tumours such as non-small cell lung cancer [12], colorectal cancer [13] and hepatocellular carcinoma [14]. At the same time, it has been shown that patients with malnutrition have a lower tolerance to adverse drug reactions during chemotherapy, which further affects the continuation of the chemotherapy process, resulting in a poorer response to chemotherapy [15]. The nutritional status during treatment is thus also a key factor in the response to chemotherapy. The prognostic nutritional index (PNI), as a simple and feasible nutritional test, can be used to some extent as an indicator of individual nutritional status and is widely used to predict the prognosis of various malignancies as well as to assess the occurrence of perioperative complications [16, 17]. Previous studies have generally used neutrophil-lymphocyte ratio (NLR) and platelet-lymphocyte ratio (PLR) traditional markers of inflammation to evaluate the prognosis of patients with gastric cancer [18–20]. However, there are few studies using SII combined with PNI to evaluate the chemosensitivity to sintilimab immunotherapy combined with XELOX chemotherapy in advanced gastric cancer patients.

In this study, we evaluated the predictive value of pre-treatment SII-PNI score on chemosensitivity and prognosis in patients with locally advanced gastric cancer who are treated with the combination of XELOX and sintilimab, in order to determine the optimal parameters for predicting survival and clinical response to immunotherapy.

## Materials and methods

### Study design and participants

This is a prospective clinical study of sintilimab immunotherapy combined with XELOX chemotherapy for locally advanced gastric cancer in the Fourth Hospital of Hebei Medical University from March 2020 to July 2021. This trial was registered at ChiCTR. gov. cn: ChiCTR2000030414. All patients were informed about the adverse effects accompanying therapies and they all signed informed consent forms. All procedures performed in studies involving human participants were in accordance with the ethical standards of the institutional and/or national research committee and with the 1964 Declaration of Helsinki and its later amendments or comparable ethical standards. The study design was approved by the Ethics Committee of the Fourth Hospital of Hebei Medical University (approval number: 2019125).

The following inclusion criteria were applied: (1) age at 18–75 years; (2) gastric adenocarcinoma confirmed by histopathology and cT3/4aN + M0 evaluated by computed tomography (CT) and laparoscopy; (3) the Eastern Cooperative Oncology Group(ECOG) activity status score of ≤ 2 points; (4) no prior chemotherapy or immunotherapy before neoadjuvant chemotherapy; (5) adequate organ function(including: bone marrow, liver, heart, kidney, etc.) with normal tests to tolerate chemotherapy; (6) no concurrent serious immune disorders or malignancies. Patients were excluded if they presented with the following: (1) difficulty in self-administering oral medication due to gastrointestinal obstruction; (2) received prior antitumor therapy, including chemotherapy, radiation, targeted therapy, or immunotherapy; (3) allergic reactions to the medication used in this study; (4) complications by severe uncontrolled infection or other serious uncontrolled concomitant disease, moderate or severe renal injury; (5) missing data; and (6) serious comorbidities identified by the investigators that could compromise patient safety or study completion.

### Chemotherapy regimen

Patients received XELOX (oxaliplatin 130 mg/m^2^ via IV infusion on day 1 with CAP 1000 mg/m^2^ oral twice daily, postprandially on the 1st to the 14th day) for four cycles (21 days/cycle). At the same time, sintilimab was administered intravenously over 1 h at a dose of 2 mg/kg every 21 days.

Four weeks after the completion of four cycles of sintilimab combined with XELOX neoadjuvant chemotherapy, the patients were evaluated with the objective response rate and resectability by CT, and the standard D2 lymph node dissection gastrectomy was performed. According to the standards formulated by the Japanese Gastric Cancer Society, three surgeons with at least 10 years of experience in radical gastrectomy for gastric cancer carried out tumor resectability assessment and performed the surgery. Patients underwent another four cycles of XELOX treatment 1 month postoperatively.

### Assessments

The tumour response is assessed according to the criteria established by the TRG grading scale (AJCC/CAP criteria) [21]. TRG 0 is defined as no residual tumour cells found microscopically on multiple consecutive sections. TRG 1 is defined as the presence of only small clusters of tumour cells that can be observed under the plasma membrane. TRG 2 is defined as fibrosis within the tumour lesion and the observation of fragmented residual tumour cells. TRG3 was defined as a lesion with little to no fibrosis and no change in the number of tumour cells. In this study, responses were categorized into TRG 3 and non-TRG 3 (including: TRG 2, TRG 1 and TRG 0).

### Definitions and follow-up

Peripheral venous blood samples were collected in fasting state within 1 week before initiation of chemotherapy in all patients. The counts of peripheral neutrophils, lymphocytes and platelets were measured and analyzed by an automatic blood analyzer (Beckman Coulter LH750), and the levels of peripheral albumin were measured and analyzed by an automatic blood analyzer (Beckman Coulter AU5800) [22]. The definitions of PNI and SII were shown as follows: PNI = albumin (g/L) + 5 × total lymphocyte counts (10^9^/L); SII = platelet × neutrophil/lymphocyte counts [22].

In this study, PD-L1 overexpression was expressed as combined positive score (CPS) > 1. The definition standard of CPS is as follows: the percentage of positive living tumor cells (any intensity of partial or complete membrane staining) and positive lymphocytes and macrophages (any intensity of membrane or cytoplasm staining) in all living tumor cells, and the results are expressed by 0–100 values (when the calculation results exceed 100, the final results are calculated according to 100) [23].

All patients are recommended to be followed up every 3 months for the first 2 years and every 6 months after 2 years. Follow-ups include telephone consultations, outpatient visits and inpatient visits. Hospital investigations include CT of the chest, abdomen and pelvis, as well as oesophagogastroduodenoscopy (EGD) and tumour markers. In this study, the deadline for follow-up was July 31, 2021. Progression-free survival (PFS) was measured from the time of treatment initiation to clinical or radiographic progression, or death from any cause.

### Statistical analyses

SPSS version 21.0 and GraphPad Prism 5.01 were utilized to perform statistical analyses. The receiver operating characteristic (ROC) curves for discriminating patients with PD from those with non-PD were carried out to get the optimal cut-off values for SII and PNI with the highest Youden’s index. Survival analysis was performed using the Kaplan–Meier method. Univariate and multivariate analyses were investigated by the Cox proportional hazards regression model. The hazard ratio (HR) and 95% confidence interval (CI) were used to assess relative risks. Spearman correlation analysis was used to evaluate the relationship between PNI and SII. *P* values < 0.05 were considered as statistically significant.

## Results

### Patients’ demographic information and tumor characteristics

**Fig. 1:**
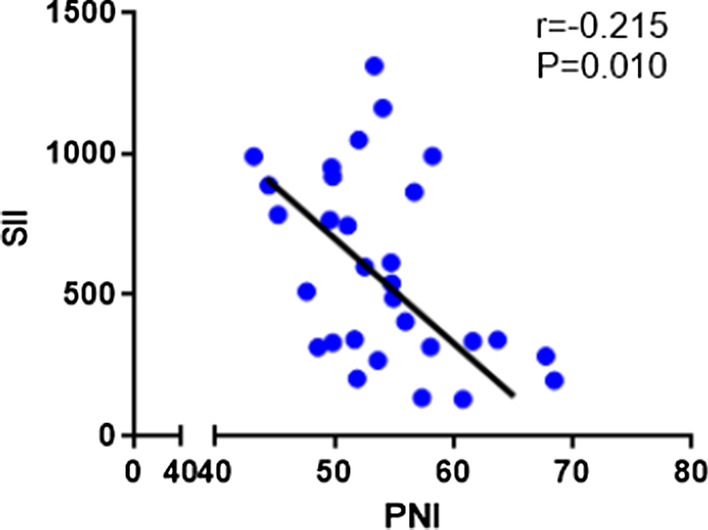
Correlation analysis between SII and PNI

This study prospectively included 30 patients with locally advanced gastric cancer. Patient demographic information and tumor characteristics are summarized in Table 1. There were 18 males (60.00%) and 12 females (40.00%). The median age of the patient was 62 years old, ranging from 30 to 75. The pathological type was poorly differentiated in 24 cases (80.00%), while moderately or well differentiated in 6 cases (20.00%). The SII and PNI ranged from 129.0 to 1311.2 and 43.2 to 68.5, respectively. The median values of pre-treatment SII and PNI were 591.5 and 54.1, respectively. Meanwhile, the two systemic indices SII and PNI had close negative correlation (r = − 0.215, *P* = 0.010; Fig. 1).

**Table 1:** Patient and tumor characteristics

Patient demographic information/tumor characteristics	Case (%)
Sex	
Male	18 (60.00)
Female	12 (40.00)
Age (years)	
< 60	7 (23.33)
≥ 60	23 (76.67)
ECOG performance status	
0	19 (63.33)
1	8 (26.67)
2	3 (10.00)
Tumor size (cm)	
< 5.0	16 (53.33)
≥ 5.0	14 (46.67)
Differentiation	
Poor	13 (43.33)
Moderately or well	17 (56.67)
Lesion site	
Cardia	10 (33.33)
Stomach	6 (20.00)
Gastric antrum	10 (33.33)
Whole stomach	4 (13.33)
cT stage	
T3	8 (26.67)
T4	22 (73.33)
PD-L1 overexpression(CPS)	
< 1	11 (36.67)
≧ 1	19 (63.33)

### Optimal cut-off values of SII and PNI before neoadjuvant chemotherapy

**Fig. 2:**
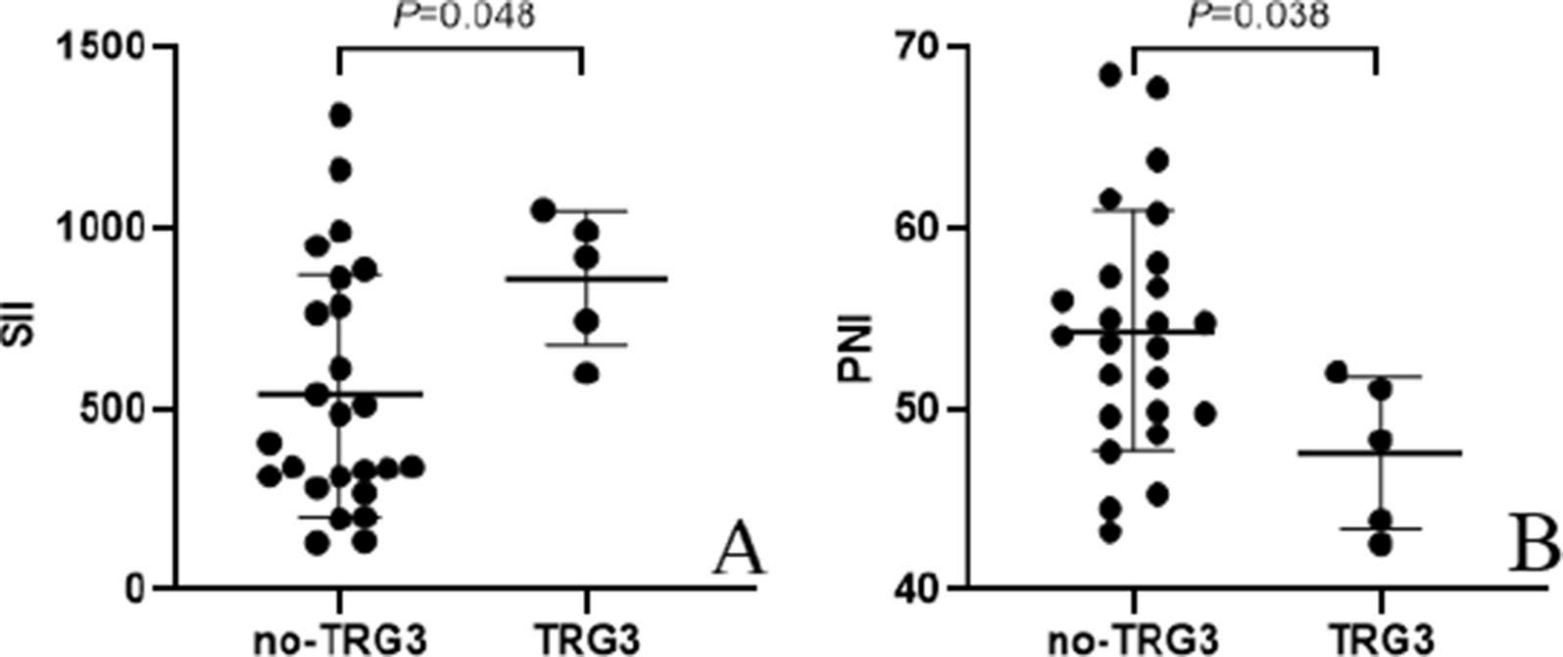
Relationship between tumor response and the SII (**A**)/PNI (**B**)

The whole group of the 30 patients, the mean SII and PNI in the 25 patients with non-TRG3 were 537.8 ± 334.8 and 54.3 ± 6.6, respectively. Meanwhile, the mean SII and PNI in the 5 patients with TRG3 were 860.0 ± 185.6 and 48.6 ± 3.1, respectively. The SII in TRG3 patients was significantly higher than that in non-TRG3 patients (*P* = 0.048), while the PNI in TRG3 patients was lower than that in non-TRG3 patients (*P* = 0.038) (Fig. 2).

**Fig. 3:**
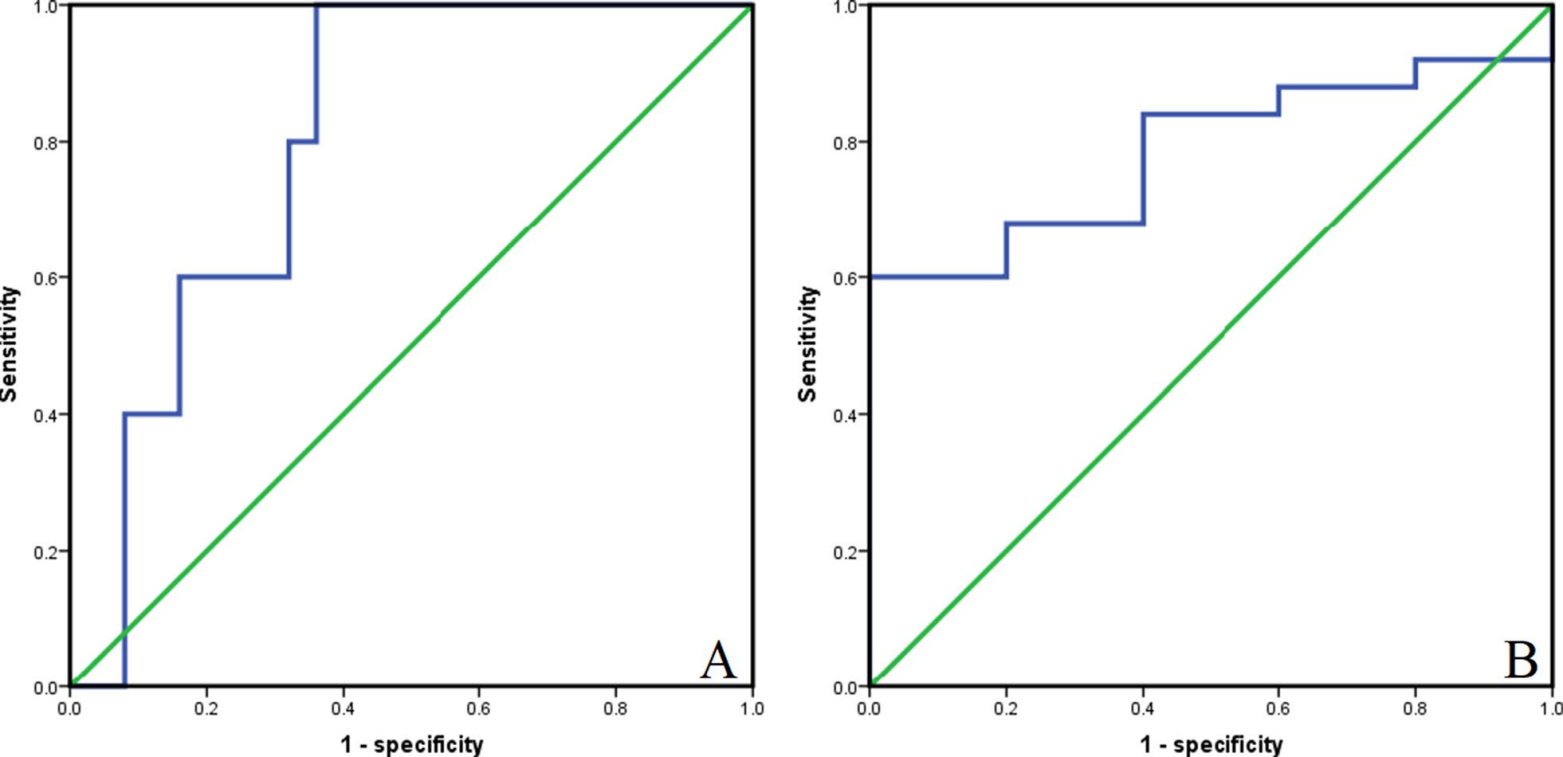
ROC curves for discriminating patients with TRG3 and those with non-TRG3 according to values of the SII (**A**) and PNI (**B**)

In order to determine the cut-off value of continuous variables, we constructed the ROC curve and calculated the AUC to evaluate the predictive ability of SII and PNI in distinguishing TRG3 and non-TRG3 patients. The optimal cut-off value of SII was 568.5 [AUC = 0.800, 95% CI 0.635–0.965, *P* = 0.037], and the corresponding sensitivity was 1.000 and specificity was 0.640. The optimal cut-off value of PNI was 52.7 [AUC = 0.784, 95% CI 0.614–0.954, *P* = 0.048], with the corresponding sensitivity of 0.600 and specificity of 1.000 (Fig. 3). According to the optimal cut-off values of SII and PNI, the patients were divided into three group: score of 2 (n = 5), high SII (≥ 568.5) and low PNI (≤ 52.7); score of 1 (n = 13), either high SII or low PNI; score of 0 (n = 12), no high SII nor low PNI.

### The relationship between SII-PNI score and neoadjuvant chemotherapy response

All patients received 4 cycles of sintilimab combined with XELOX neoadjuvant chemotherapy and completed enhanced abdominal and pelvic CT scan after treatment. All patients underwent radical surgery for gastric cancer. According to the TRG classification criteria (AJCC/CAP criteria), 10 patients (33.33%) were TRG 0, 9 patients (30.00%) were TRG 1, 6 patients (20.00%) were TRG 2, and 5 patients (16.67%) were TRG 3. The SII-PNI score was significantly lower in patients with non-TRG3 than in those with TRG3 (*p* = 0.017) (Table 2).

**Table 2:** Relationship between tumor response and the SII-PNI score

Tumor response	SII-PNI score (%)			*P* value
	0 (n = 12)	1 (n = 13)	2 (n = 5)	
Non-TGG3 (n = 25)	11 (31.1)	12 (47.6)	2 (14.3)	0.017
TGG3 (n = 5)	1 (11.1)	1 (22.2)	3 (66.7)	

### Relationship between SII-PNI score and prognosis

All patients were followed up, and the median follow-up period was 13 months (3.5–19.3 months). The 1-year progression-free survival (PFS) was 75.00%, and the median PFS (mPFS) was 10.4 months (95% CI 6.2–14.9 months). The mPFS of patients with SII-PNI score of 0, 1, and 2 were 11.8, 9.4, and 6.3 months, respectively, and the difference between the three groups was significant (all *P* < 0.001). Multivariate analysis showed that tumor response (*P* = 0.002), SII-PNI score (*P* = 0.003), tumor size (*P* = 0.020), and differentiation (*P* = 0.009) were independent risk factors (Table 3).

**Table 3:** Univariate and multivariate analyses of the clinicopathological characteristics for PFS

Independent factor	Univariate analysis			Multivariate analysis		
	Hazard ratio	95% CI	*P* value	Hazard ratio	95% CI	*P* value
Sex			0.532			
Female	1.000	Reference				
Male	1.176	0.625–1.621				
Age (years)			0.243			
< 60	1.000	Reference				
≥ 60	1.242	0.913–1.751				
Tumor response			0.002			0.002
Non-TRG3	1.000	Reference		1.000	Reference	
TRG3	6.379	3.231–9.127		4.429	2.742–7.421	
SII-PNI score			0.001			0.003
0	1.000	Reference		1.000	Reference	
1	1.889	1.346–3.692		1.967	0.875–2.928	
2	3.576	2.328–6.246		2.758	1.167–4.276	
Tumor size (cm)			0.002			0.020
< 5.0	1.000	Reference		1.000	Reference	
≥ 5.0	2.512	1.351–4.392		2.059	1.431–4.821	
Differentiation			0.001			0.009
Poor	1.000	Reference		1.000	Reference	
Well	2.256	1.256–3.344		3.499	1.874–7.748	

## Discussion

At present, the treatment methods for locally advanced gastric cancer mainly include radical resection, radiotherapy, chemotherapy, targeted therapy, and immunotherapy [24]. In recent years, neoadjuvant chemotherapy combined with immunotherapy has garnered more attention. The KEYNOTE-811 trial found that immunotherapy pembrolizumab combined with trastuzumab and chemotherapy increased the objective response rate of HER2-positive gastric cancer by 22.7%, and the incidence of adverse events is less [25]. However, some studies have found that not all patients benefit from this therapy, with about 30% of the disease progression after neoadjuvant chemotherapy combined with immunotherapy [26]. Although PD-L1 expression has been suggested to be useful in predicting treatment response, an optimal patient selection strategy regarding PD-L1 has not yet been established. For these patients, this combination treatment not only increases the relevant medical costs, but also may weaken the immune system and delay the optimal timing of surgery. Therefore, before neoadjuvant chemotherapy combined with immunotherapy is carried out for patients with locally advanced gastric cancer, a simple indicator to accurately predict the therapeutic effect will be beneficial to the formulation and selection of individualized treatment regimens. Due to a relatively poor response rate in this population, identification of such a predictor has been both a major challenge and a priority in this field.

Previous studies have explored the relationships between inflammatory response and the occurrence and development of malignant tumors. Moreover, the nutritional status of patients is also an important factor affecting the progression of tumors [27, 28]. SII, which is composed of peripheral blood neutrophil, lymphocyte, and platelet counts, can comprehensively measure systemic inflammation. SII alone has been proven to predict prognosis of various malignant tumors [11]. Meanwhile, most studies investigated clinical values of PNI alone in patients with gastric cancer and other malignant tumors [17]. To the best of our knowledge, we were the first to combine SII and PNI to establish SII-PNI score as a new scoring system for predicting tumor response and prognosis in patients with locally advanced gastric cancer after neoadjuvant chemotherapy combined with immunotherapy.

Tumor response is one of the most important prognostic factors in patients with locally advanced gastric cancer undergoing neoadjuvant chemotherapy. Unfortunately, it is difficult to predict tumor response using clinical pathological information before treatment. Therefore, we focus on SII and PNI to overcome the challenges related to prediction of tumor response. Numerous studies have shown that SII can be used to predict whether breast cancer has pathological complete remission after neoadjuvant chemotherapy and the prognosis of patients [29, 30]. PNI is also widely used in clinical practice to evaluate the efficacy, adverse reactions, and prognosis of neoadjuvant chemotherapy in esophageal cancer, lung cancer, and other tumors [16, 17]. However, the use of systemic inflammatory response indicators combined with nutritional status indicators, and the development of a SII-PNI scoring system for predicting the efficacy of neoadjuvant chemotherapy and evaluating the prognosis of patients has not yet been reported. In this study, we analyzed the relationship between SII-PNI score and the efficacy of XELOX combined with sintilimab in patients with locally advanced gastric cancer after neoadjuvant chemotherapy. The results of this study showed that the SII-PNI score was closely related to the efficacy of chemotherapy. Lower SII-PNI scores were associated with better efficacy of chemotherapy combined with immunotherapy. This suggested that the SII-PNI score could be a candidate marker for predicting tumor response in locally advanced gastric cancer patients after neoadjuvant chemotherapy. This is a cost effective and simple test to identify patients who are most likely to respond and therefore deliver high value care. There are proteomic and genetic analyses currently underway to identify clinically useful predictive markers, however these are currently limited due to limited commercial availability.

We also assessed the relationship between the SII-PNI score and prognosis. In this study, we analysed the risk factors that may influence patients with locally advanced gastric cancer receiving neoadjuvant chemotherapy with XELOX in combination with sintilimab and found that the SII-PNI score was an independent risk factor for patient survival and prognosis. The mPFS of patients with SII-PNI score of 0, 1, and 2 were 11.8, 9.4, and 6.3 months, respectively. This indicated that with the increase of SII-PNI score, the prognosis of patients became worse, and the disease recurrence was more likely to occur. Possible mechanisms for the SII-PNI to predict prognosis are as follows: (1) A higher SII-PNI score indicates an increase in neutrophil count and/or platelet count relative to lymphocyte count. Neutrophils significantly inhibit lymphokine-activated killer cell-mediated cytotoxic effects, thereby downregulating the patient's anti-tumour cellular immune response and neutrophils also release vascular endothelial growth factor (VEGF), a pro-angiogenic factor associated with promoting tumour formation, invasion and metastasis [31, 32]. In addition, the increased platelet count promotes tumour growth through excessive secretion of platelet-derived growth factor (PDGF) and VEGF, and also promotes the adhesion of the tumour system to blood vessels, which further facilitates the metastasis of metastatic cells [33, 34]. (2) An increase in SII-PNI score also indicates a relative decrease in lymphocytes, suggesting that the patient may be immunosuppressed or deficient, thus promoting the progression of tumour progression and thus affecting the patient's prognosis [35, 36]. (3) The decrease in serum albumin levels in the body reflects the poor nutritional status of the patient, who is in a state of malnutrition. The poorer the nutritional status of the patient, the lower the immunity of the body, which in turn leads to disease progression [37].

It is noteworthy that a few limitations of current research also exist. First, this prospective study was conducted in a single center with a small sample size (n = 30). Second, this study only selected XELOX combined with sintilimab for analysis. Given the potentially significant clinical benefit demonstrated by our results, larger, multi-center prospective studies investigating different treatment regimens are urgently needed to confirm our findings. It may be interesting to also look into safety and probability of significant adverse events, and if the SII-PNI is able to predict this too.

## Conclusions

In conclusion, our study demonstrates that SII-PNI score was useful in predicting the efficacy response and survival outcome of locally advanced gastric cancer patients after being treated with combined neoadjuvant chemotherapy of XELOX regimen and immunotherapy of sintilimab. These findings may be beneficial to the formulation of therapeutic strategies and clinical risk stratification to avoid unnecessary toxicity and resource abuse in patients who are unlikely to benefit from treatment.

## Data Availability

The datasets used and/or analyzed during the current study are available from the corresponding author on reasonable request.
